# Supplementary material for: Mapping the evolution of fertility support policies in China: A content and instrumental analysis
Source: PLoS One. 2025 Oct 9;20(10):e0332137. doi: 10.1371/journal.pone.0332137 (PMC12510515; doi:10.1371/journal.pone.0332137)
Supplement: S1 Appendix — (ZIP) [file pone.0332137.s001.zip › S1 Appendix. 226 original policy documents/10-国家人口和计划生育委员会、中央社会治安综合治理委员会办公室、财政部、人力资源和社会保障部关于创新流动人口服务管理体制推进流动人口计划生育基本公共服务均等化试点工作的指导意见(FBM-CLI-4-138786).doc]

国家人口和计划生育委员会、中央社会治安综合治理委员会办公室、财政部、人力资源和社会保障部关于创新流动人口服务管理体制推进流动人口计划生育基本公共服务均等化试点工作的指导意见

**发布部门：** [国家人口和计划生育委员会(含国家计划生育委员会)(已撤销)](https://www.pkulaw.com/law/chl?Aggs.IssueDepartment=60304) [中央社会治安综合治理委员会](https://www.pkulaw.com/law/chl?Aggs.IssueDepartment=7060222) [财政部](https://www.pkulaw.com/law/chl?Aggs.IssueDepartment=60210) [人力资源和社会保障部](https://www.pkulaw.com/law/chl?Aggs.IssueDepartment=60236) [机构沿革](javascript:void(0))

**发文字号：**人口流管〔2010〕69号

**发布日期：**2010.09.21

**实施日期：**2010.09.21

**时效性：** [失效](https://www.pkulaw.com/law/chl?Aggs.TimelinessDic=02)

**效力级别：** [部门工作文件](https://www.pkulaw.com/law/chl?Aggs.EffectivenessDic=XE0303)

**法规类别：** [计划生育管理](https://www.pkulaw.com/law/chl?Aggs.Category=09705)

**失效依据：**[国家卫生健康委员会关于宣布失效第三批委文件的决定](javascript:TMP(316932,0,'chl'))

国家人口和计划生育委员会、中央社会治安综合治理委员会办公室、财政部、人力资源和社会保障部关于创新流动人口服务管理体制推进流动人口计划生育基本公共服务均等化试点工作的指导意见
（人口流管〔2010〕69号）

各省、自治区、直辖市人口计生委、综治委、财政厅（局）、人力资源社会保障厅（局）：
　　为贯彻落实《[流动人口计划生育工作条例](https://www.pkulaw.com/chl/f6b2c35d55b7a40ebdfb.html)》和中央关于推动流动人口服务管理体制创新、推进基本公共服务均等化的精神和要求，现就在部分城市开展创新流动人口服务管理体制、推进流动人口计划生育基本公共服务均等化试点工作提出以下指导意见：
　　**一、**充分认识创新流动人口服务管理体制、推进流动人口计划生育基本公共服务均等化试点工作的重要意义
　　流动人口计划生育基本公共服务均等化是指流动人口在现居住地获得与户籍人口同等的宣传倡导、计划生育、优生优育、生殖健康、奖励优待等方面的基本公共服务。创新流动人口服务管理体制、推进流动人口基本公共服务均等化，切实保障流动人口合法权益，是稳定低生育水平、统筹解决人口问题的迫切要求；是推进人口城镇化、统筹城乡区域协调发展的客观要求；是构建和谐社会，实现社会公平正义的有效途径；是加快政府职能转变，建立服务型政府的现实需要。
　　当前，我国正处于城镇化、工业化和城乡区域一体化快速发展的新时期，人口流动迁移规模不断扩大。流动人口为流入地经济社会发展做出了重要贡献。同时，大规模人口流动迁移，也对各级政府的社会管理和公共服务带来了巨大挑战。由于传统的以户籍为依托的社会管理体制难以适应新形势的需要，城市社区建设滞后，流动人口计划生育服务管理经费投入不到位、工作人员不足，国家规定的流动人口免费计划生育技术服务未能完全落实，流动人口难以享受到与户籍人口同等的计划生育基本公共服务，其合法权益得不到可靠保障。如果这些问题得不到有效解决，不仅低生育水平难以稳定，还会衍生出一系列社会问题。各地务必站在统筹解决人口问题的高度，充分认识创新流动人口服务管理体制、推进流动人口计划生育基本公共服务均等化工作的重要性，进一步增强责任感和紧迫感，通过试点，积极探索，积累经验，因地制宜，抓出实效。

　　**二、**指导思想、工作目标和基本原则
　　（一）指导思想。
　　以科学发展观为指导，按照统筹解决人口问题，维护社会公平、构建和谐社会的要求，坚持“公平对待、服务至上、合理引导、完善管理”的方针，以创新流动人口服务管理体制为核心，通过统筹规划，分步实施，系统推进，建立健全流动人口计划生育基本公共服务运行机制，切实保障流动人口的基本权利和发展权利，促进人口与经济社会的协调和可持续发展。
　　（二）工作目标。
　　通过组织实施试点、有序推进和逐步深化，到2012年，试点城市计划生育基本公共服务覆盖全部流动人口，流动人口与当地户籍人口计划生育基本公共服务差距明显缩小，基本建立运转高效、保障有力的流动人口计划生育基本公共服务均等化运行机制，加快流动人口市民化进程。初步建立适应城镇化、促进城乡一体化发展的流动人口服务管理新体制。
　　（三）基本原则。
　　1．以人为本。把维护广大流动人口的切身利益和合法权益，推进基本公共服务均等化，作为推动流动人口服务管理体制创新的根本出发点和落脚点。坚持人本管理理念，不断满足流动人口对计划生育基本公共服务均等化的需求，寓管理于服务之中，通过加强服务实现管理目标。
　　2．统筹推进。试点城市要发挥各方优势，加强人口计生与社会治安综合治理、公安、教育、民政、财政、人力资源社会保障、卫生等相关部门的政策衔接，积极探索创新体制机制，整合部门管理力量和信息资源，形成政府主导、公共财政为主体的计划生育基本公共服务供给模式。真正实现流动人口计划生育基本公共服务均等化，积极促进其他各项基本公共服务均等化，全面提升流动人口均等化服务水平。
　　3．重点突破。试点城市要结合本地实际，制定切实可行的改革目标、任务和工作规划，在领导体制、综合决策、居住证管理、社区登记、信息共享、投入保障、服务网络、服务拓展、城乡统筹等一个或多个方面，有针对性地进行改革创新，推动试点，积极探索行之有效的流动人口服务管理模式。

　　**三、**主要任务
　　（一）建立健全流动人口工作统筹管理、综合决策体制。
　　建立党政领导负责的人口（或流动人口）工作领导协调常设机构，切实加强对流动人口工作的统筹协调。加强流动人口计划生育服务管理政策与户籍管理、劳动就业、教育、医疗、社会保障、住房等方面的政策制度的衔接和协调，制定和完善均等化服务相关公共政策，形成部门协同推进流动人口基本公共服务均等化工作合力。
　　实行城乡统一的人口登记管理。进一步深化户籍管理制度改革，着力破除城乡分割的二元社会管理体制。健全出租房屋、企事业单位、人力资源市场、暂住人口、流动育龄妇女、流动党团员等流动人口登记管理制度。逐步将公共服务享受资格与户籍制度剥离，稳步推行居住证制度，建立健全城乡统一的人口登记管理制度。
　　（二）全面推进流动人口计划生育基本公共服务均等化。
　　1．宣传倡导服务。强化宣传教育的针对性和有效性，积极开展关爱流动人口的主题宣传服务活动，制作和发放免费宣传品，普及人口和计划生育政策法规以及优生优育、生殖保健科普知识，保障流动人口免费参加有关人口和计划生育法律知识以及生殖健康知识普及活动，引导流动人口树立科学、文明、进步的婚育观念。流动人口计划生育政策知晓率达到90%以上。
　　2．计划生育技术服务。组织从事计划生育技术服务的机构，免费为流动人口提供国家规定的计划生育基本服务项目，按规定为流动人口提供免费的孕情环情检查。流动人口在流入地接受免费服务的比例逐年提高，2012年达到95%以上。流动已婚育龄妇女在现居住地免费接受孕检率达到90%以上。深化药具服务管理体制改革，实现流动人口免费药具全覆盖。流动人口免费药具获得率达到90%以上。
　　3．优生优育服务。加强优生优育科普知识宣传和普及，为流动育龄人口免费提供咨询服务，组织开展婴幼儿早期启蒙活动，将流动人口纳入出生缺陷一级预防、免费孕前优生健康检查等项目中，流动人口在流入地享有与户籍人口同等的计划生育优质服务。流动已婚育龄妇女获得免费孕前优生咨询等项目服务率达到80%以上。做好宣传倡导，防止发生非医学需要的胎儿性别鉴定和非医学需要的选择人工终止妊娠行为，保证母婴安全。
　　4．生殖健康服务。加强避孕节育、生殖健康科普宣传，提供生殖健康咨询指导，为流动人口提供必要的随访服务。将流动育龄妇女纳入当地生殖健康项目服务。做好流动育龄妇女生殖健康档案管理，落实住院分娩、B超检查、计划生育手术等实名登记制度，及时更新相关信息，并实行网络化管理。
　　5．奖励优待服务。采取多种措施，建立流动人口计划生育利益导向机制。对自觉落实长效避孕措施的流动育龄夫妻，可按规定给予奖励；流动人口晚婚晚育或在现居住地施行计划生育手术的，享受与户籍人口同等的休假等待遇。
　　在扎实做好流动人口计划生育基本公共服务均等化的同时，认真落实企业职工基本养老保险和流动就业人员基本医疗保障关系的转移接续办法，切实加强新生代农民工服务管理。加强相关政策协调，促进流动人口获得子女教育、技能培训、医疗、社保、文化体育设施、公共福利等其他各项基本公共服务，优先解决实行计划生育的流动人口在城市的落户问题，加快流动人口融入城市社会进程。
　　（三）完善流动人口基本公共服务网络体系。
　　试点城市要在编制当地城市发展规划、制定公共政策、建设公用设施等方面，统筹考虑流动人口对计划生育公共服务的需求，完善流动人口计划生育管理机构和服务网络。依托城市基层社会治安综治中心、农民工综合服务中心、流动人口服务中心等综合工作平台，实现流动人口“一条龙”服务、“一站式”管理。
　　切实增强城市社区流动人口服务力量。加强社区便民维权工作，促进和谐社区建设。立足社区，整合社会资源，将流动人口计划生育服务管理纳入城市社区工作人员的职责。根据流动人口服务管理需要，合理配备社区计划生育工作人员，加强对从业人员的教育培训。在流动人口集中的社区、企业、集贸市场等成立流动人口计划生育协会，实行自我管理、自我教育、自我服务；发挥基层计划生育协会会员和社区志愿者的作用，在社区开展流动人口计划生育宣传与生育关怀工作，引导、帮助流动人口增强实行计划生育的自觉性，提高维护自身合法权益的能力。
　　（四）建立健全流动人口综合服务管理信息系统。
　　充分利用人口计生、统计、公安、人力资源社会保障等部门管理资源，建立统一的全员流动人口信息采集、更新和动态管理制度，开展流动人口变动趋势的预警监测和综合分析，全面掌握流动人口生存发展状况，为党委政府科学决策和流动人口享受基本公共服务提供信息支撑。依托国家和省级流动人口服务管理信息系统，构建和完善试点城市流动人口综合服务管理信息平台，实现流动人口信息跨部门、跨系统、跨地区共享。

　　**四、**保障措施
　　（一）切实加强对试点工作的领导。
　　国家人口计生委、中央综治办、财政部、人力资源社会保障部等部门成立推进流动人口计划生育基本公共服务均等化试点工作协调小组，负责试点工作的组织实施和统筹协调，加强对试点城市工作的指导，开展试点评估，及时总结推广试点经验。试点城市所在的省级相关部门要加强对试点工作的协调指导，帮助解决试点工作中出现的问题。各省（区、市）也可参照本意见，组织具备条件的城市开展相关试点工作。试点城市要将流动人口基本公共服务均等化试点工作纳入地方社会经济体制改革的总体规划、纳入党委、政府的重要议事日程，成立由党政领导牵头的试点工作组织领导机构，周密部署，统筹推进。
　　（二）建立流动人口基本公共服务均等化经费保障机制。
　　要按照与户籍人口同等投入标准，认真研究和确定流动人口计划生育基本公共服务项目和开支，将流动人口计划生育工作所需经费纳入本地财政预算予以保障。
　　（三）强化双向管理。
　　试点城市要加强与流动人口流出地的信息沟通和服务管理协作配合。流动人口所在户籍地，要落实源头管理责任，积极配合流入地做好相关工作。流入地和流出地在生育政策执行、计划生育服务、信息交换、违法生育处理等方面要认真做好协同工作，切实推进流动人口计划生育双向管理和区域协作，促进全国“一盘棋”。
　　（四）强化考核评估。
　　国家建立试点城市工作绩效评估制度，对经过评估成绩突出的试点城市，在国家地方转移支付经费和药具经费调拨等方面给予倾斜。有关流动人口计划生育工作项目优先在试点城市实施。试点城市要建立流动人口计划生育基本公共服务均等化考核评估机制，健全考核评估体系和科学考评方法，细化工作职责、目标和任务，确保试点工作有序开展、取得实效。
　　各地要注意研究试点过程中出现的新情况、新问题，积极探索和总结经验，及时报送试点工作进展情况。

国家人口和计划生育委员会
中央社会治安综合治理委员会办公室
财政部
人力资源和社会保障部
二○一○年九月二十一日

© 北大法宝：（[www.pkulaw.com](https://www.pkulaw.com/)) 专业提供法律信息、法学知识和法律软件领域各类解决方案。北大法宝为您提供丰富的参考资料，正式引用法规条文时请与标准文本核对。
欢迎查看所有[产品和服务](https://www.pkulaw.com/helps/2.html)。法宝快讯：[如何快速找到您需要的检索结果？](https://www.pkulaw.com/helps/70.html)[法宝](https://www.pkulaw.com/helps/69.html)V6有何新特色？

扫描二维码阅读原文

**原文链接：**<https://www.pkulaw.com/chl/4ee0f00b959f5960bdfb.html>
